# Integrating Placebo Effects in General Practice: A Cross-Sectional Survey to Investigate Perspectives From Health Care Professionals in the Netherlands

**DOI:** 10.3389/fpsyt.2021.768135

**Published:** 2022-01-12

**Authors:** Rosanne M. Smits, Dieuwke S. Veldhuijzen, Henriët van Middendorp, Marianne J. E. van der Heijden, Monique van Dijk, Andrea W. M. Evers

**Affiliations:** ^1^Health, Medical and Neuropsychology Unit, Leiden University, Leiden, Netherlands; ^2^Pediatric Immunology and Rheumatology, Wilhelmina Children's Hospital, Utrecht, Netherlands; ^3^Leiden Institute for Brain and Cognition (LIBC), Leiden University, Leiden, Netherlands; ^4^Department of Internal Medicine, Nursing Science, Erasmus Medical Center, Rotterdam, Netherlands; ^5^Department of Pediatric Surgery, Erasmus Medical Center - Sophia Children's Hospital, Rotterdam, Netherlands; ^6^Department of Psychiatry, Leiden University Medical Center, Leiden, Netherlands

**Keywords:** placebo effects, clinician communication, attitudes and acceptability, cross-sectional survey, nurses, health care professional

## Abstract

**Objectives:** Placebo effects, beneficial treatment outcomes due to non-active treatment components, play an important role in the overall treatment response. To facilitate these beneficial effects it is important to explore the perspectives of health care professionals (HCPs) on the integration of placebo effects in clinical care. Three themes were investigated: knowledge about placebo effects and factors that contribute to these, frequency of placebo use, and attitudes toward acceptability and transparency of placebo use in treatment.

**Methods:** A cross-sectional survey, according to the Checklist for Reporting Results of Internet E-Surveys guidelines and STrengthening the Reporting of OBservational Studies in Epidemiology (STROBE), was conducted in the Netherlands in 2020. The survey was conducted in two samples: a (nested) short survey in 78 nurses during working shifts (sample 1) and an extended online survey in 47 general HCPs e.g., medical psychologists, oncologists, surgeons (sample 2).

**Results:** Respondents from both samples reported to be somewhat or quite familiar with placebo effects (24.0 and 47.2%, respectively). From the six placebo mechanisms that were presented, mind-body interaction, positive expectations, and brain activity involved in placebo effects were rated as the most influential factors in placebo effects [F_(5,119)_ = 20.921, *p* < 0.001]. The use of placebo effects was reported in 53.8% (*n* = 42) of the nurses (e.g., by inducing positive expectations), and 17.4% of the HCPs (*n* = 8 reported to make use of pure placebos and 30.4% of impure placebos (*n* = 14). Attitudes toward placebo use in treatment were acceptant, and transparency was highly valued (both up to 51%).

**Conclusions:** The findings from this study address knowledge gaps in placebo effects in practice and provide insights in attitudes toward the integration of placebo effects from HCPs. Altogether, integrating these findings may potentially optimize treatment outcomes.

## Introduction

Placebos are inert substances that inherently lack properties to induce any effect (1). Placebo effects, however, can induce beneficial treatment outcomes due to non-active treatment components. These components can entail learning mechanisms (e.g., classical conditioning and expectancy learning) or contextual factors (e.g., empathic communication and trust) (2–4). In the literature, a distinction is often made between placebo use and the use of placebo effects. In terms of placebo use, research often addresses pure placebos (without active pharmacological properties, such as sugar pills) and impure placebos (with pharmacological properties but not for the specific symptoms, such as antibiotics for viral infections) (1, 5–7). In terms of placebo effects, the use of learning mechanisms and contextual effects are mentioned that induce beneficial effects (1, 4). Frequency of placebo use (pure and impure) by health care practitioners (HCP) have been studied broadly and vary between 41 and 99% across countries (e.g., Switzerland, Canada, UK and the US) (5–11). Frequencies on the use of placebo effects, however, are scarce and need to be investigated further.

To learn more about the use of placebo effects in health care, it is important to include a wide range of HCPs. However, current literature mostly describe the perspectives of doctors while the perspectives of nurses are underrepresented (12, 13). Because nursing practices encompass many facets that facilitate placebo effects (e.g., empathic communication, trust) the perspectives of this group should not be missed (12–14, 16). Moreover, investigating the perspectives on the use of placebo effects in practice may help to understand how placebo effects can best be utilized in general practice.

In the present study, perspectives on placebos and placebo effects were explored in HCPs by assessing three themes: (1) knowledge about placebo effects and their attributing factors, (2) frequency of placebo use, and (3) attitudes toward acceptability and transparency for placebo use in treatment. In addition to the current literature, this study specifically includes nurses, an overlooked group of HCPs, and focuses on their perspectives on integrating placebo effects in general practice.

## Methods

### Study Design

A cross-sectional survey study was performed in the Netherlands according to the checklist Strengthening the Reporting of Observational Studies in Epidemiology (STROBE), see Supplementary Material 1 (17). The study was carried out in nurses at the Erasmus University Medical Center in Rotterdam, embedded in the WELCOME study, as approved by the Medical Ethics Review Committee (MEC-2017-1103). Due to the Covid-19 outbreak and its impact on the availability of nurses, a second sample of HCPs was added to be more in line with sample sizes from previous studies (ranging from 169 to 2018 HCPs) (5–10, 18–21). This second sample of HCPs received an extended version of the survey, as approved by the Psychology Research Ethics Committee of Leiden University (2020-04-07-A.W.M. Evers-V1-2368). See Table 1 for an overview of the sample characteristics.

**Table 1:** Sample characteristics^a^.

	**HCPs (*N* = 47)**	**Nurses (*N* = 78)**	
Years of health care experience^b^	17.3 (13.8)	14.2 (11.8)	
Age^b^	41.0 (12.0)	33.8 (11.9)	
Gender (N M:F)	11:36	21:57	
**Specialization**	**Frequency (%)**	**Specialization**	**Frequency (%)**
Psychology^c^	11 (23.4)	Intensive care	42 (53.8)
Oncology^d^^,^^e^	8 (17.0)	Medium care internal medicine	25 (32.1)
Pediatrics^c^^,^^d^^,^^e^	4 (8.5)	Medium care surgery	11 (14.1)
Surgery^d^^,^^e^	3 (6.4)		
Medical doctor (unspecialized)^d^	3 (6.4)		
Geriatrics^d^	3 (6.4)		
Maternity care^d^^,^^e^	3 (6.4)		
General practitioner^d^	3 (6.4)		
Emergency room^d^^,^^e^	2 (4.3)		
Endocrinology^e^	2 (4.3)		
Unspecified^d^	2 (4.3)		
Phlebology^d^	1 (2.1)		
Anesthesia^e^	1 (2.1)		
Urology^d^	1 (2.1)		

a *Overall completion rate was 75.4%*.

b *Mean (SD)*.

c *Psychologist*.

d *Medical doctor*.

e *Nurse*.

*HCPs, Health care professionals*.

### Recruitment and Respondents

Respondents from the first sample represent a sample of nurses from general wards and intensive care units at the Erasmus University Medical Center in Rotterdam, the Netherlands. They were recruited during or at the end of a work shift and invited to fill in the survey on a tablet. The second sample consisted of a broader range of HCPs recruited through social media platforms (LinkedIn) and the researchers' networks. The short survey (nurses) took 10 min to fill out and the extended survey (HCPs) took 15 min. The study took place on site for the nurses (on a tablet) and online for the HCPs between May and August, 2020.

### Measures

The short survey (sample 1: nurses) consisted of 7 items, and the extended survey (sample 2: HCPs) of 14 items (see Table 2). Both surveys were based on a questionnaire that was developed to explain underlying mechanisms of placebo effects and categorized in three themes (3). For current knowledge, respondents were asked about familiarity with placebo effects and nocebo effects on a 5-point Likert scale (from very unfamiliar to very familiar) and how they would explain these effects (free-text entry). To rate the influence of important placebo factors (e.g., positive expectations, patient-practitioner relationship, mind-body interaction, social-observational learning, brain activity related to positive expectations, and classical conditioning), respondents estimated each influence on treatment outcomes on a numerical slider (i.e., 0% not important, 50% somewhat important, 100% very important) (3). Furthermore, respondents were asked about placebo use (sample 1) and pure and impure placebo use (yes/no questions) (sample 2). A third theme was added in the extended survey to assess attitudes toward acceptability and transparency of placebo use with varying answer categories (i.e., in case of psychological complaints, a cold, chronic diseases, terminal diseases, never correct, or always correct). Multiple answers were possible. See Table 2 for an overview of the survey and samples.

**Table 2:** Overview and results of survey questions (*N* = 125).

							**Sample 1 (*N* = 78)**	**Sample 2 (*N* = 47)**
**1**	**Current knowledge of placebo effects**	Not at all	Slightly	Somewhat	Quite	Very much		
	How familiar are you with the placebo effect?	1 (0.8)	19 (15.2)	30 (24.0)	59 (47.2)	16 (12.8)	✓	✓
	How familiar are you with the nocebo effect?	10 (21.3)	15 (31.9)	6 (12.8)	13 (27.7)	3 (6.4)		✓
		**Strongly disagree**	**Disagree**	**Neutral**	**Agree**	**Strongly agree**		
	Do you believe that placebo effects can improve treatment outcomes?	0 (0.0)	1 (0.8)	31 (24.8)	69 (55.2)	24 (19.2)	✓	✓
	Do you believe that nocebo effects (negative expectations) can deteriorate treatment outcomes?	1 (0.8)	6 (4.8)	58 (46.4)	44 (35.2)	16 (12.8)	✓	✓
	Do you want to learn more about placebo effects?	0 (0.0)	6 (4.9)	22 (18.0)	82 (67.2)	12 (9.8)	✓	✓
	Can you describe an example of when you experienced a placebo effect in a patient?	Free text entry^b^					✓	✓
	Can you describe an example of when you experienced a nocebo effect in a patient?	Free text entry^b^						✓
	How would you explain the placebo effect to a patient?	Free text entry^b^						✓
	How much do you think these factors influence treatment outcomes in %?	M		SD	95%CI			
	• Positive expectations	74.5		19.0	[71.4–77.6]		✓	✓
	• Good relationship between practitioner and patient	73.5		17.4	[70.0–77.0]		✓	✓
	• Mind-body interaction	75.1		15.1	[71.9–78.2]		✓	✓
	• Seeing or hearing positive experiences from other patients	69.2		17.6	[66.0–72.4]		✓	✓
	• Brain activity related to positive expectations	73.7		18.0	[71.0–76.4]		✓	✓
	• Classical conditioning (the body learns from medication)	59.9		19.7	[56.5–63.3]		✓	✓
**2**	**Frequency of placebo use**	Yes	No					
	Have you ever made use of placebo effects?	42 (53.8)	36 (46.2)				✓	
	Have you ever made use of **pure** placebos?^c^	8 (17.4)	38 (82.6)					✓
	Have you ever made use of **impure** placebos?^c^	14 (30.4)	32 (69.6)					✓
**3**	**Acceptability of placebo use**							
	Attitudes toward acceptability of placebo use	See Figure 2						✓
	Attitudes toward transparency of placebo use	See Figure 3						✓

*^a^N, (%)*.

b *An example from the most common answers will be provided*.

c *N = 46*.

### Procedure

After providing informed consent, the respondents of sample 1 filled in background characteristics followed by introductory information about placebo and nocebo effects. In sample 2, a differentiation between pure and impure placebos was made and additionally explained (see Supplementary Material 2 for the provided descriptions). Subsequently, respondents were presented with the survey.

### Statistical Analysis

Data was analyzed using IBM SPSS Statistics (version 25). Data were summarized using percentages and cross-tabulations. Percentages of perceived influence of placebo factors were compared on a within-subject level in a repeated measures ANOVA. Pairwise comparisons were Bonferroni-corrected. Assumptions were checked, and corrections were made for sphericity violations (Huynh-Feldt correction) (22). Partial eta squared (ηp^2^) was reported for effect size (23). A significance level of <0.05 was set as statistically significant.

Responses from free text entry fields were handled based on the grounded theory methodology (24). The answers that were most frequently mentioned were used as in-text examples. Missing data were handled based on listwise deletion.

## Results

### Sample Characteristics

#### Placebo Knowledge: Likert Scales

Sample characteristics are described in Table 1. Most of the respondents from both samples reported to be somewhat or quite familiar with placebo effects (*M* = 3.56, *SD* = 0.93 on a 5-point scale). The sample of HCPs seemed less familiar with nocebo effects (*M* = 2.66, *SD* = 1.27 on a 5-point scale). See Table 2 for an overview of all numbers and percentages of familiarity with placebo and nocebo effects, treatment benefits, and interests in learning about placebo effects.

#### Placebo Knowledge: Perceived Influence of Placebo Mechanisms

**Figure 1:**
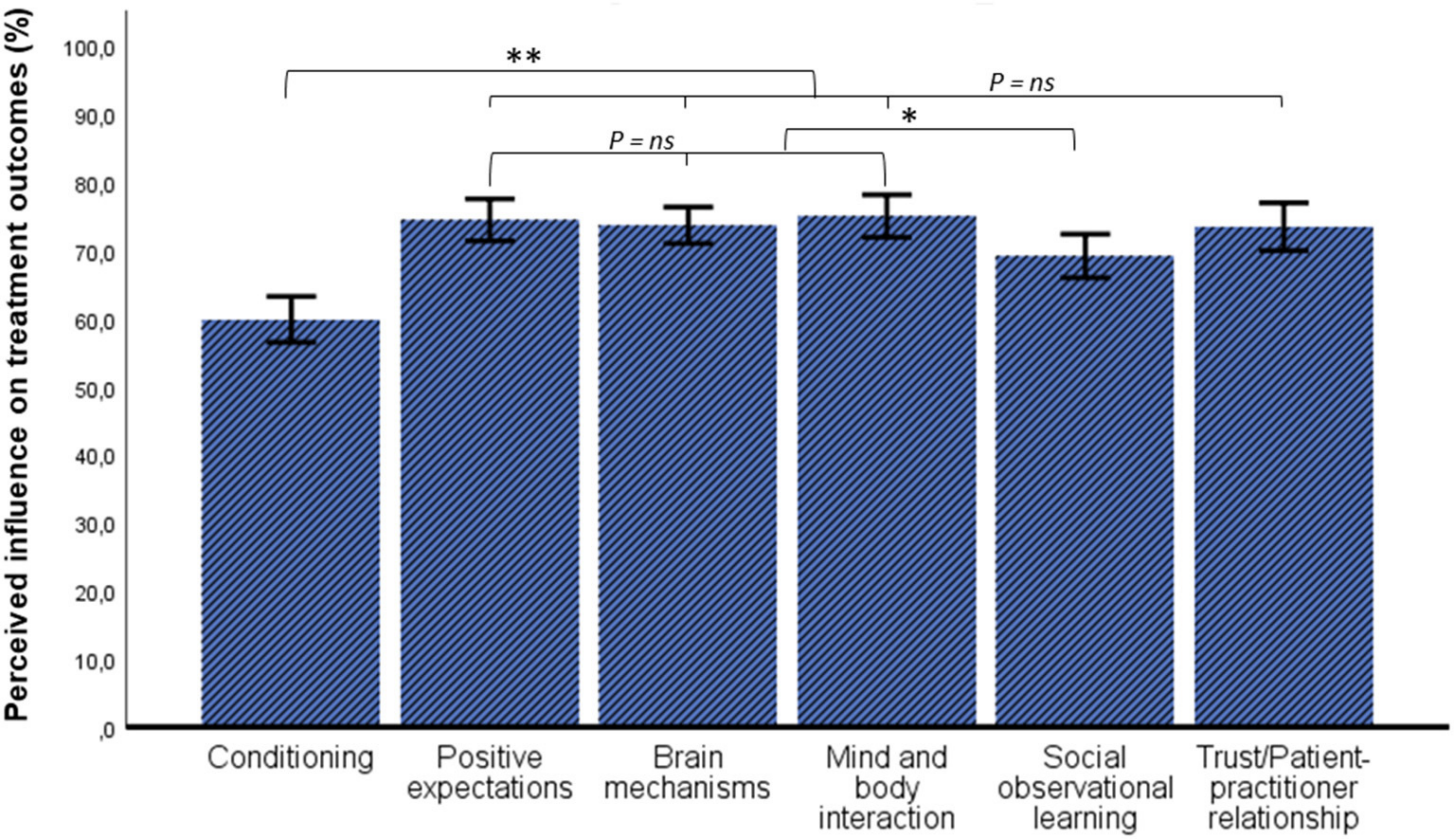
Ratings of perceived influence of placebo factors in treatment outcomes. Error bars: 95% CI, *p < 0.05, **p < 0.001.

To understand how respondents rated the influence of specific placebo factors, Bonferroni-corrected pairwise comparisons were carried out. A significant difference was found between perceived influence of the different placebo factors on treatment outcomes [*F*_(5,119)_ = 20.921, *p* < 0.001, ηp^2^ = 0.145]. Pairwise comparisons indicated that conditioning was rated significantly lower than all other factors. Positive expectations, brain mechanisms and mind-and-body interaction were rated significantly more influential than social learning and conditioning. All factors were rated above 50% (Figure 1).

### Placebo Knowledge: Free Text Entry

#### Example of Placebo Use

The majority of the respondents (74 of 125; 59%) were able to provide an example. The most common example was the administration of paracetamol (acetaminophen) to induce sleep.

#### Example of Nocebo Use

Twenty-five out of 47 respondents (53%) were able to provide an example. The most common example described how negative expectations influence treatment outcomes adversely.

#### Explaining Placebo Effects to Patients

Of the 47 HCPs, 43 (91%) were able to provide an example. The most common examples were based on mind-and-body interaction, positive expectations, and brain activity induced by placebo effects. Six respondents (12.8%) reported to restrain from explaining placebo effects, because they thought this would negate the positive effects.

### Attitudes Toward Acceptability and Transparency

**Figure 2:**
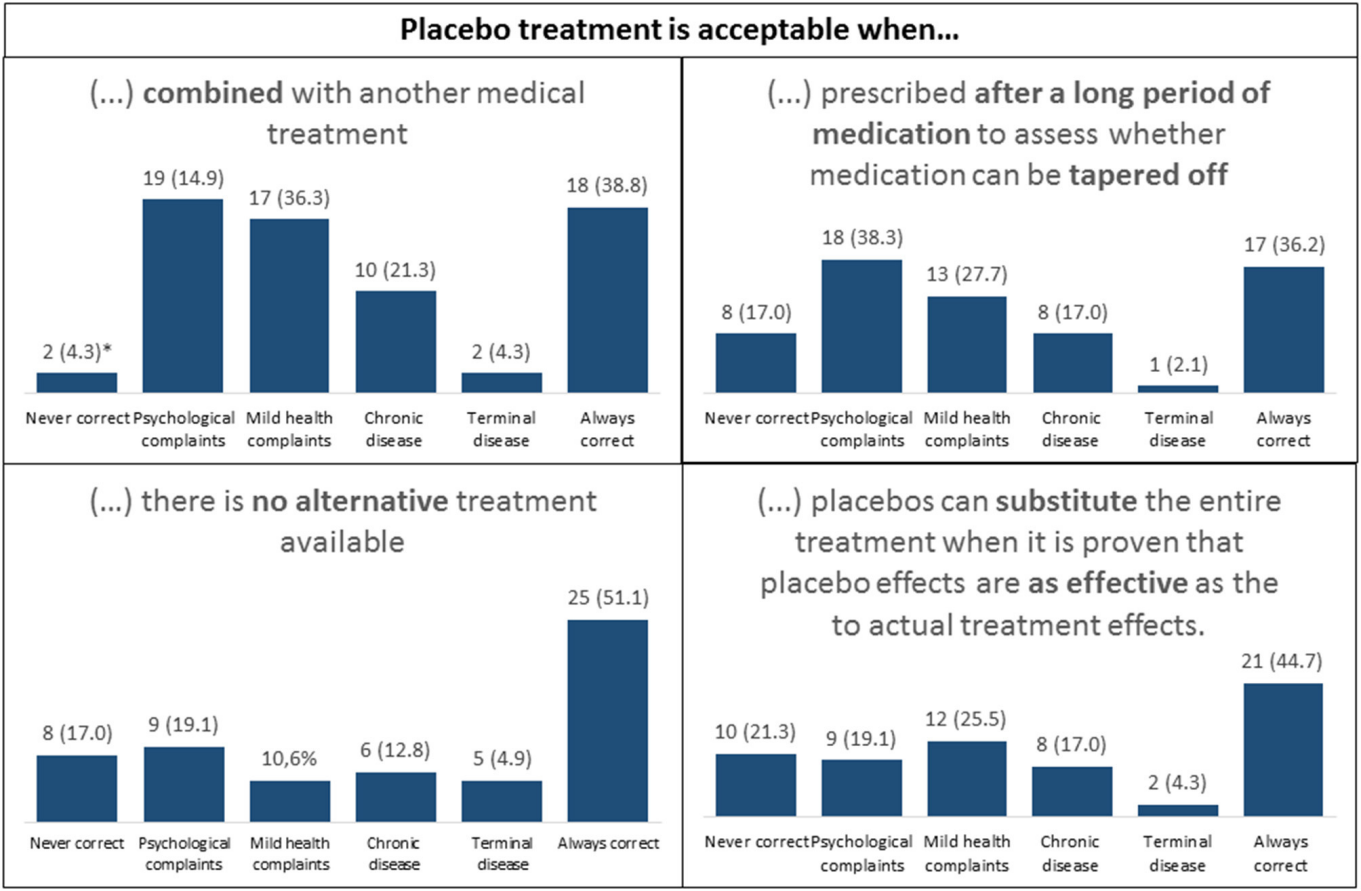
Outcomes of placebo acceptability scores in different scenarios.*N (%).

**Figure 3:**
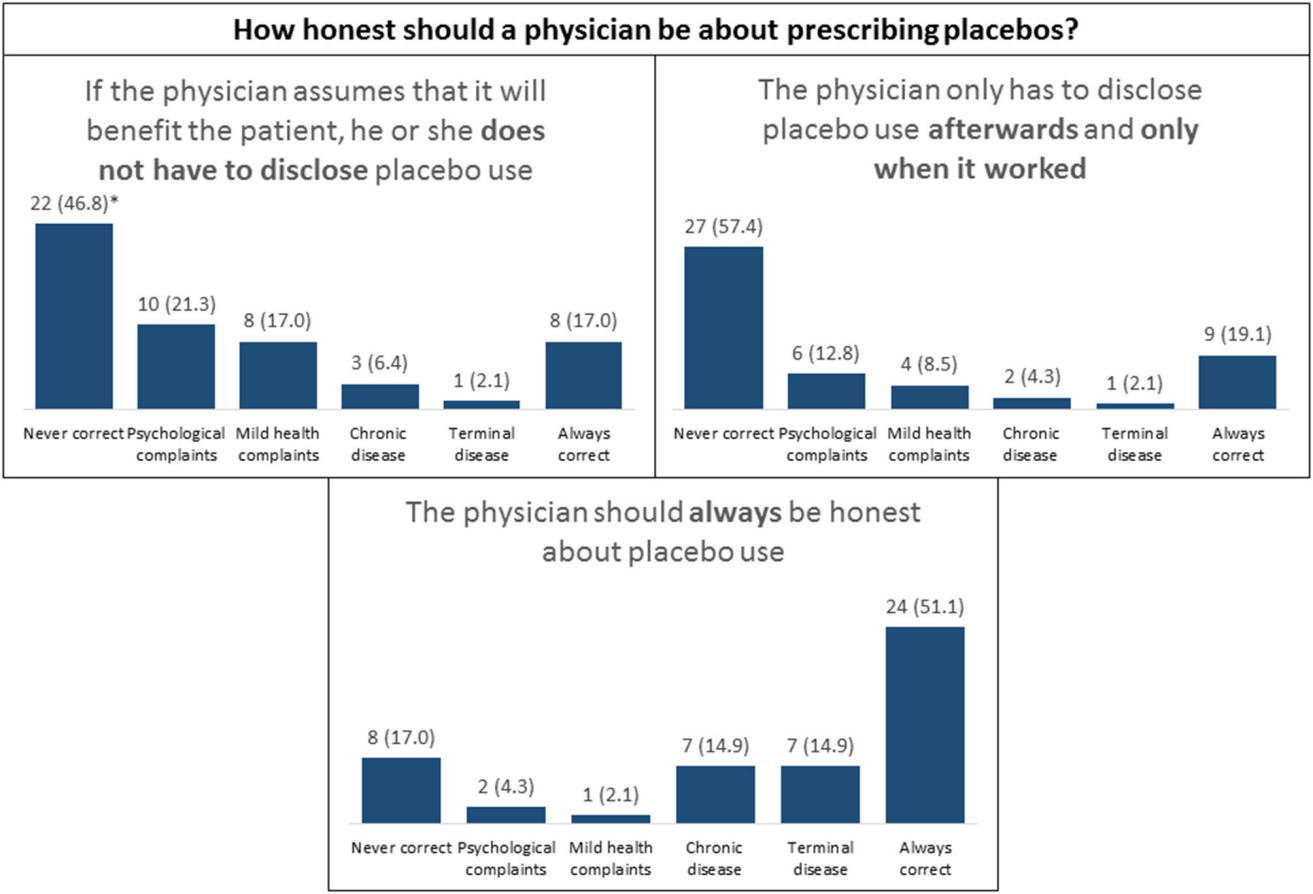
Outcomes of placebo transparency scores in different scenarios. *N (%).

For acceptability, we found the highest percentages for “always correct,” followed by “acceptable for psychological complaints” and “acceptable for mild health complaints.” For transparency, the highest percentages were found in the category “never correct” (up to 51%), even though 21% indicated that deception was correct if the placebo had worked (see Figures 2, 3).

## Discussion

The present study explored perspectives of nurses and other health care professionals (HCPs) on the integration of placebo effects in clinical care based on three themes: knowledge about placebo effects and factors that contribute to these, frequency of placebo use, and attitudes toward acceptability and transparency of placebo use in treatment. Initially we aimed to only include a sample of nurses, but due to the impact of Covid-19 we extended the sample with other HCPs such as doctors and psychologists. Overall, the benefits of placebo effects and factors that contribute to treatment outcomes were well-understood by the respondents. The potential harm of nocebo effects, however, was less known. The use of placebos (pure and impure) was reported by approximately half of the respondents. Moreover, respondents were predominantly accepting of the (transparent) use of placebo effects.

Results from the first theme, placebo knowledge, indicated that respondents were overall familiar with placebo effects. With regards to nocebo effects, respondents seemed to be less familiar, also supported by the finding that only half of the respondents could describe an example thereof in the free-text entries. Moreover, results from the free-text fields indicated a misconception about deception, namely that explaining placebo effects would negate their effects and respondents therefore refrained from explaining these. These findings are insightful since the current trend in placebo research is leaning toward the direction of open-label placebos, where placebo effects can be elicited without deception, which seemed to be unknown in this study and other studies (3, 5, 21, 25). Placebo factors were perceived as influential in treatment with scores of 50% or higher, with mind and body-interaction, brain mechanisms, and positive expectations receiving the highest scores. Noteworthy, in a previous study that assessed placebo explanations based on similar factors, it was also found that positive expectations and brain mechanisms were rated as the most preferred explanations (3). Moreover, previous studies that included positive suggestions as impure placebos techniques revealed that approximately half of the respondents (general practitioners) use this technique almost daily (7, 10, 20). In line with previous studies, this present study highlights two insights, namely that respondents are knowledgeable about placebo mechanisms that involve positive expectations and brain mechanisms, and that these mechanisms can serve as helpful tools to explain placebo effects. Additionally, most respondents from our sample also indicated to be interested in learning more about placebo effects.

The second theme focused on the frequency of placebo use. Overall, the use of placebos reported in this study (53.8%) was considerably lower compared to previous studies from Germany (88%), Poland (80%), and the UK (97%) (10, 18, 20). Moreover, results from our study indicated that both samples make use of impure placebos, for example by the use of paracetamol to induce sleep, which was the most common example described. In sample 2, we found that impure placebos were more frequently used than pure placebos (30 vs. 17%). The latter percentages were also lower than the results of a systematic review about pure and impure placebo use (45 vs. 76%) (6, 26). A reason for this discrepancy may pertain to Dutch health care legislation, where physicians are obligated to inform patients about the medication that is prescribed, and placebo use may therefore be much lower than in other countries (15).

Finally, HCPs were generally acceptant toward placebo use in treatment, with the highest acceptance in subgroups of psychological or mild complaints and the lowest in case of terminal disease. Transparency was highly valued, with highest percentages in the category “never correct” for scenarios that described the use of deception, which is also in line with previous studies in general practitioners (8, 27), psychiatrists (11), and orthopedic surgeons (28).

Limitations were sample size and suboptimal inclusion because of Covid-19 (6). Even though our research aim was initially to include a homogeneous sample of nurses, we had to extend our sample to health care professionals in general, due to the great amount of pressure on nurses in the first line of care. In future research, nurses should be more included in samples and insights should be gathered about how HCPs want to be educated and trained about the use (and misuse) of placebo and nocebo effects in practice. Additional questions about nocebo effects (i.e., nocebo explanations) could be developed and implemented to gain insights in knowledge gaps, and explore how negative expectations can be harnessed to prevent adverse treatment outcomes.

## Conclusion

HCPs in the Netherlands (nurses, psychologists, and doctors) report to use placebos and placebo effects in practice. Respondents indicated to be interested in learning about placebo effects and were acceptant of their (transparent) use. Moreover, HCPs evaluated placebo factors as influential in treatment, such as positive expectations, brain mechanisms, and mind-and body-interaction, which may be addressed in medical education or in communication with patients. Altogether, integrating these findings may potentially optimize treatment outcomes.

## Data Availability Statement

The original contributions presented in the study are included in the article/Supplementary Materials, further inquiries can be directed to the corresponding author/s.

## Ethics Statement

The studies involving human participants were reviewed and approved by Medical Ethical Committee, Leiden University Medical Centre, Leiden, The Netherlands Medical Ethical Committee, Erasmus Medical Centre, Rotterdam, The Netherlands. The patients/participants provided their written informed consent to participate in this study.

## Author Contributions

RS, DV, HM, MH, MD, and AE: primary data collection. RS: performing analysis and writing first draft of the manuscript. All authors contributed to the conception or design of the study and/or interpretation of the data as well as critically reviewing and editing the manuscript.

## Funding

This work was supported by grants of the Dutch Arthritis Foundation (ReumaNederland 16-3-401), the European Research Council (ERC Consolidator Grant ERC-2013-CoG-617700), and the Dutch Organization for Scientific Research (NWO-Vici grant 01 6.V I CL770. L52), granted to AE.

## Conflict of Interest

The authors declare that the research was conducted in the absence of any commercial or financial relationships that could be construed as a potential conflict of interest.

## Publisher's Note

All claims expressed in this article are solely those of the authors and do not necessarily represent those of their affiliated organizations, or those of the publisher, the editors and the reviewers. Any product that may be evaluated in this article, or claim that may be made by its manufacturer, is not guaranteed or endorsed by the publisher.
