# Supplementary material for: Integrating Placebo Effects in General Practice: A Cross-Sectional Survey to Investigate Perspectives From Health Care Professionals in the Netherlands
Source: Front Psychiatry. 2022 Jan 12;12:768135. doi: 10.3389/fpsyt.2021.768135 (PMC8790122; doi:10.3389/fpsyt.2021.768135)
Supplement: Supplementary file 2 [file Data_Sheet_2.pdf]

S2 File. Information provided about placebo and nocebo effects

S2: Descriptions provided about placebo and nocebo effects

Information provided about placebo and nocebo effects	
Total sample (N=125)	"Offering a patient a glass of milk and telling him or her that this will make him or her sleep better is an example of placebo use that can lead to a placebo effect. Placebo effects can have a large (positive) influence on the treatment and therefore be very useful. With the nocebo effect, expectations have a negative effect on the treatment."
Health care professionals (N=47)	"Pure placebos are treatments that have no pharmacological effect (e.g. sugar pills)." "Impure placebos are treatments with pharmacological effects, but not on the condition being treated (e.g. antibiotics in viral infections or vitamins)."
